# Regulator of Chromosome Condensation 1-Domain Protein DEK47 Functions on the Intron Splicing of Mitochondrial *Nad2* and Seed Development in Maize

**DOI:** 10.3389/fpls.2021.695249

**Published:** 2021-08-02

**Authors:** Shi-Kai Cao, Rui Liu, Aqib Sayyed, Feng Sun, Ruolin Song, Xiaomin Wang, Zhihui Xiu, Xiaojie Li, Bao-Cai Tan

**Affiliations:** ^1^Key Laboratory of Plant Development and Environment Adaptation Biology, Ministry of Education, School of Life Sciences, Shandong University, Qingdao, China; ^2^Key Laboratory of Cell Activities and Stress Adaptations, Ministry of Education, School of Life Sciences, Lanzhou University, Lanzhou, China; ^3^Agricultural Genomics Institute, Chinese Academy of Agricultural Sciences, Shenzhen, China

**Keywords:** mitochondria, regulator of chromosome condensation 1-domain protein, intron splicing, *nad2*, complex I, maize, seed development

## Abstract

In flowering plants, mitochondrial genes contain approximately 20–26 introns. Splicing of these introns is essential for mitochondrial gene expression and function. Recent studies have revealed that both nucleus- and mitochondrion-encoded factors are required for intron splicing, but the mechanism of splicing remains largely unknown. Elucidation of the mechanism necessitates a complete understanding of the splicing factors. Here, we report the identification of a regulator of chromosome condensation 1 (RCC1)-domain protein DEK47 that is required for mitochondrial intron splicing and seed development in maize. Loss of function in *Dek47* severely arrests embryo and endosperm development, resulting in a *defective kernel (dek)* phenotype. DEK47 harbors seven RCC1 domains and is targeted to mitochondria. Null mutation of DEK47 causes a deficiency in the splicing of all four *nad2* introns, abolishing the production of mature *nad2* transcript and resulting in the disassembly and severely reduced activity of mitochondrial complex I. In response, the expression of the alternative oxidase *AOX2* is sharply increased in *dek47*. These results indicate that *Dek47* is required for the splicing of all the *nad2* introns in mitochondria, and essential for complex I assembly, and kernel development in maize.

## Introduction

Mitochondria are the sites of oxidative phosphorylation (OXPHOS) and essential metabolic pathways, such as the beta oxidation, the citric acid cycle, amino acid breakdown, and apoptosis (Millar et al., 2011; Spinelli and Haigis, 2018). Owing to gene migration to nucleus, approximately 95% of the proteins required for normal functions of mitochondria are encoded by the nuclear genome, leaving a mere 5% encoded by the mitochondrial genome (Richardson et al., 2013). Furthermore, expression of the mitochondrial genes is strictly regulated by the nucleus which is primarily executed at the post transcriptional level *via* RNA terminal maturation, RNA cleavage, RNA editing, and intron splicing.

Intron splicing is a major event of mitochondrial gene expression in plants, in which the noncoding regions (introns) in mRNAs are excised and the coding sequences (exons) are ligated (Bonen, 2008). Based on tertiary structures, introns are divided into group I and group II (Bonen and Vogel, 2001). Group I introns contain a P1-P9 catalytic center and do not possess a consensus splice site, whereas group II introns consist of six domains (DI-DVI) extending from a central hub and possess a “GU-AG” consensus splice site (Ferat and Michel, 1993; Lambowitz and Belfort, 2015). Almost all of the organellar introns in higher plants belong to group II. Canonical group II introns possess the ribozyme activity that catalyzes the self-splicing. In bacteria and yeast, group II intron splicing is facilitated by intron encoded proteins or the maturase that is encoded by the intron itself (Lehmann and Schmidt, 2003; Toro et al., 2007). The maturase is suggested to bind with a high affinity and specificity to its cognate intron sequence to assist the folding of the intron into a catalytic configuration. Then, the intron performs self-splicing. During the evolution of land plants, however, group II introns have lost nearly all the maturase genes except the *matR* gene imbedded in the *nad1* intron 4 (Wahleithner et al., 1990; Sultan et al., 2016). Compounded with accumulating genetic alterations in the intron sequences, group II introns in mitochondria have lost the self-splicing capability. Consequently, the splicing of introns in mitochondria is facilitated by various RNA-binding proteins. As mitochondrial gene expression is essential to plant growth and development, defects in intron splicing render severe impacts on plant growth and development (Brown et al., 2014; Hammani and Giege, 2014), which facilitates the genetic and molecular dissection of the splicing factors. Studies in the last 10 years have shown that RNA-binding proteins from distinct protein families are required for the splicing of mitochondrial and chloroplast introns. These protein families include the chloroplast RNA splicing and ribosome maturation (CRM) proteins (Ostersetzer et al., 2005; Asakura et al., 2008; Zmudjak et al., 2013; Chen et al., 2019), nuclear-encoded maturases (Nakagawa and Sakurai, 2006; Keren et al., 2009, 2012; Cohen et al., 2014; Zmudjak et al., 2017; Shevtsov-Tal et al., 2021), RNA DEAD-box helicases (Asakura et al., 2012), mitochondrial transcription termination factors (Hsu et al., 2014), plant organellar RNA recognition (PORR) proteins (Colas des Francs-Small et al., 2012), pentatricopeptide repeat (PPR) proteins (de Longevialle et al., 2007; Koprivova et al., 2010; Xiu et al., 2016), and regulator of chromosome condensation (RCC1) family proteins (Kuhn et al., 2011).

The RCC1-like domain (RLD) proteins are defined by containing one or more RLD domains (Dasso, 1993). Within the RLD domain exists tandem repeats of degenerated 51–68 amino acid residues, known as RCC1 repeats (Hadjebi et al., 2008). The human RCC1 contains seven RCC1 repeats that are folded into a seven-bladed beta-propeller fold (Renault et al., 1998; Hadjebi et al., 2008). Human RCC1 is a critical cell cycle regulator with functions in nucleo-cytoplasmic transport, cell cycle control, and chromosome binding (Renault et al., 1998). In animals, RCC1-domain proteins have been shown to mediate diverse biological processes, such as interaction with proteins and lipids, and enzyme inhibition (Hadjebi et al., 2008). In *Arabidopsis thaliana*, 24 putative RCC1 family proteins have been identified, among which four [UVR8, RCC1/UVR8/GEF-like 3 (RUG3), tolerant to chilling and freezing 1 (TCF1), sensitive to ABA 1 (SAB1)] have been characterized (Brown et al., 2005; Kuhn et al., 2011; Ji et al., 2015, 2019). UVR8, dual-targeted to cytosol and nucleus, is involved in UV-protective responses for plant survival in sunlight (Brown et al., 2005; Rizzini et al., 2011). RUG3 in mitochondria is required for the splicing of *nad2* introns 2 and 3, and modulates ROS homeostasis (Kuhn et al., 2011; Su et al., 2017). TCF1 localized in the nucleus is required for modulating lignin biosynthesis to promote freezing tolerance and cold acclimation (Ji et al., 2015). SAB1 that is dual-localized in cytoplasm and nucleus negatively regulates ABI5 in ABA signaling (Ji et al., 2019). In maize, 31 RCC1-domain proteins have been identified, but none have been characterized thus far.

Here, we show that the RCC1 domain protein DEK47 is essential to maize kernel development. We prove that DEK47 is required for the splicing of the four introns of *nad2* in mitochondria. Loss of DEK47 function impairs the splicing and causes a deficiency of Nad2 protein, which results in reduced assembly of mitochondrial complex I, enhanced alternative pathway, and arrested maize kernel development.

## Materials and Methods

### Plant Materials and Growth Conditions

The *Zea mays Mutator*-insertion allele of *dek47* (*dek47-1*) was isolated from the UniformMu population (McCarty et al., 2005). The second allele of *dek47* (*dek47-2*) was obtained from the Maize EMS-induced Mutant Database population (Maize EMS-induced Mutant Database)^1^ (Lu et al., 2018). *Dek47-1* (±) heterozygotes were identified by PCR with gene-specific primer *Dek47*-R2 and *Mutator*-specific *Mu*-TIR8 primer. The *A. thaliana* T-DNA insertion lines *rug3-1* (SALK_092071) and *rug3-2* (GABI_466G01) were obtained from the Arabidopsis Biological Resource Center. The maize plants were grown in the experimental field under natural conditions in Qingdao and Sanya, China. Arabidopsis and tobacco (*N. benthamiana*) plants were grown in pots under 22°C with a 16-h photoperiod and 25°C with a 12-h photoperiod, respectively.

### Light Microscopy of Cytological Sections

For light microscopy, the immature kernels of *dek47-1* and WT at 8 and 14 days after pollination (DAP) were sectioned from segregating ears for paraffin sections. Sections of the maize kernels were fixed in 4% (w/v) paraformaldehyde, dehydrated in ethanol, infiltrated and embedded in paraffin, de-paraffinized, stained with Safranin O, and imaged with a stereo microscope (Carl-Zeiss, Jena, Germany) as described previously (Zhang et al., 2013).

### Subcellular Localization of DEK47

The full-length coding sequence of *Dek47* was amplified using high-fidelity DNA polymerase KOD plus Neo (TOYOBO) and cloned into vector pENTR™/D-TOPO^®^ (pENTR^™^ Directional TOPO^®^ Cloning Kits, Invitrogen), performed LR recombination reaction (Gateway^®^ LR Clonase^™^ II Enzyme Mix, Invitrogen) with the binary vector pGWB5. For subcellular localization, DEK47-GFP was transiently expressed in tobacco epidermal cells using MitoTracker Red CMXRos (Invitrogen) as a marker of mitochondria with a working concentration of 100 nm, as described previously (Liu et al., 2013). Fluorescence signals were observed under a ZEISS LSM 880 confocal microscope (Carl-Zeiss, Jena, Germany).

### RNA Extraction, RT-PCR, and qRT-PCR

For RNA isolation, total RNA was isolated from the embryo and endosperm of immature kernels of *dek47-1* and WT at 12 DAP using the RNeasy Mini Kit (Qiagen, Germany). The RNA was treated with RNase-free DNase I (NEB) to remove potential DNA contaminants. Reverse transcription-PCR (RT-PCR) and Quantitative real-time PCR (qRT-PCR) were performed according to the manufacturer’s instructions (TransGen Company, Beijing, China). RNAs were normalized against the maize *Actin* gene (*GRMZM2G126010*) or the Arabidopsis *Actin* gene *ACT2 (AT3G18780)*. Information of all the primers used in this study is listed in Supplementary Table S1.

### Analysis of Mitochondrial RNA Splicing

The maize and Arabidopsis mitochondrial RNA splicing was analyzed as described previously (Xiu et al., 2016; Weissenberger et al., 2017). This analysis was done with three biological replicates.

### BN-Page and NADH Dehydrogenase Activity Analysis

Blue native-polyacrylamide gel electrophoresis (BN-PAGE) and detection of NADH dehydrogenase activity were performed basically as described (Meyer et al., 2009). Crude mitochondria from fresh wild-type and *dek47*-1 kernels were extracted and used for BN-PAGE using a Native-PAGE sample prep kit (Invitrogen) as described previously (Chen et al., 2016). The gel was stained with Coomassie brilliant blue (CBB) 250 and incubated in the reaction buffer (0.14 mM NADH, 1.22 mM NBT, and 0.1 M Tris–HCl, pH 7.4) to detect the NADH dehydrogenase activity. 130 μg of crude mitochondrial membrane protein extracts from wild-type (WT) and *dek47-1* mutant maize kernels was loading in BN-PAGE gels.

### Immunoblot Analysis

Different quantities (2, 4, 8, and 8 μg) of crude mitochondrial membrane protein extracts from the developing kernels at 11 DAP were separated by 12.5% SDS-PAGE. After transferring to a nitrocellulose membrane (0.45 mm; Millipore), the membrane was incubated with primary antibodies using polyclonal rabbit antibodies against Nad9, Cyt*_C_*, Cyt*_C1_*, Cox2, ATPase, and AOX as described previously (Liu et al., 2020a). The HRP-conjugated goat anti-rabbit antibody (Abcam) was used as the secondary antibody. Signals were visualized on X-ray films (Kodak, Tokyo, Japan) using the ECL reagents (Pierce, Thermo Fisher Scientific, Waltham, MA, United States) according to the manufacturer’s instructions.

### Determination of Respiration Rate

The respiration rates were determined according to the previous report with modifications (Wang et al., 2015). Respiration rates were indicated by O_2_ consumption of the fresh maize kernels at 11 DAP using a Clarke-type Chlorolab II oxidative electrode (Hansatech).

### Complementation of the Arabidopsis *Rug3* With Maize *Dek47*

To test the genetic complementation of the Arabidopsis *rug3* with maize *Dek47*, the protein coding region of *Dek47* was cloned in pENTR/D-TOPO and transferred to the binary vector pGWB2 using the gateway system. This placed the *Dek47* gene under the CaMV35S promoter. This construct was delivered to the *Agrobacterium* strain EHA105 and transformed into the Arabidopsis *rug3-1* and *rug3-2* mutants.

### Yeast Two-Hybrid Analysis

The experiments were performed by using the Yeastmaker Yeast Transformation System 2 (Clontech). The *Dek47* sequence with a truncation of the N-terminal 1–40 amino acids was cloned into the pGADT7-AD and pGBKT7-BD vectors used as prey or bait, respectively. These constructs were co-transfected into Y2H Gold strain and spotted onto the DDO (SD/−Leu/−Trp) medium and QDO (SD/−Ade/-His/−Leu/−Trp) medium.

### Luciferase Complementation Imaging Assay

The coding sequences of DEK47, PPR14, PPR20, PPR-SMR1, Zm-mTERF15, and Zm-mCSF1 were cloned into pCAMBIA1300-nLUC [N-terminal luciferase (NLUC)] and pCAMBIA1300-cLUC [C-terminal luciferase (CLUC)], respectively, according to a previous report (Chen et al., 2008). The 4-week-old tobacco leaves were infiltrated with *Agrobacterium* strain EHA105 containing different combinations of NLUC and CLUC fusion constructs. The combination of ZmMORF8-NLUC and ZmMORF1-CLUC was used as a positive control (Liu et al., 2020b). The luciferase signals were imaged using the Lumazone FA Pylon2048B system. The experiments were carried out with at least three replicates.

## Results

### Phenotypic and Genetic Characterization of *Dek47-1*

**Figure 1:**
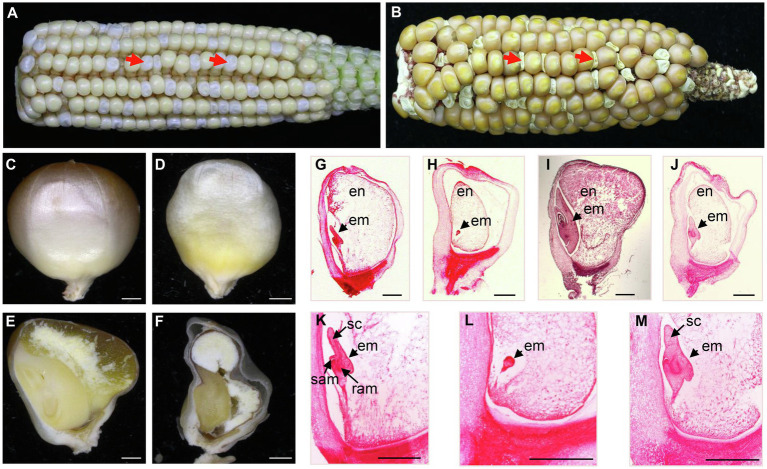
Mutant *dek47-1* arrests early in maize seed development. **(A)** The recessive *dek47-1* mutant segregated in a self-pollinated ear at 17 days after pollination (DAP). Arrows point to *dek47-1* mutant kernels. **(B)** Maturity ear of self-pollinated *dek47-1* heterozygotes. Arrows point to *dek47-1* mutant kernels. **(C,D)** Embryo side of mature kernels of wild-type **(C)** and *dek47-1* **(D)** kernels at 30 DAP. **(E,F)** Dissection of mature wild-type **(E)** and *dek47-1* **(F)** kernels at 30 DAP. **(G-M)** Paraffin sections of the wild-type (WT) and *dek47-1* kernels at 8 and 14 DAP. Wild-type kernels at 8 DAP **(G,K)** and 14 DAP **(I)**; the *dek47-1* kernels at 8 DAP **(H,L)** and 14 DAP **(J,M)**. Arrows indicate the embryo. en, endosperm; em, embryo; ram, root apical meristem; sam, shoot apical meristem; and sc, scutellum. Scale bars = 1 mm.

To reveal the function of the RCC1-like proteins in maize, we analyzed the *Mu* insertional mutants in RCC1-like genes in the UniformMu population in the W22 genetic background (McCarty et al., 2005). A *Mu* insertion line (UFMu-06802) in an RCC1-like gene (*GRMZM2G114748*) was chosen for further study because its progeny segregated *defective kernels* (*dek*), named *dek47-1*. As shown in Figures 1A,B, developing *dek47-1* kernels were white and watery at 17 DAP and collapsed at maturity. The ratio between the *dek47-1* and wild-type (WT) kernels in the self-pollinated ears was approximately 1:3 (Figures 1A,B), suggesting that *dek47-1* is a monogenic recessive mutation. The *dek47-1* kernels remained smaller and whiter than its siblings at 30 DAP (Figures 1C,D).

Maize embryo development is characterized by four stages, pre-embryo, transition, coleoptilar, and late embryogenesis. Endosperm development is characterized by four stages, coenocyte, cellularization, differentiation, and maturation (Olsen, 2001). Sectioning of the mature seeds showed that the *dek47-1* kernel contained an abnormal embryo and a small endosperm (Figures 1E,F). The comparison between the mutant and WT in the same ear indicated that the embryo and endosperm development was inhibited in *dek47-1*. When the wild-type embryo developed into the coleoptilar stage at 8 DAP (Figures 1G,K), the d*ek47-1* embryo reached the pre-embryo stage (Figures 1H,L). When the WT embryo reached the late embryogenesis stage (Figure 1I), the *dek47*-*1* embryo appeared to just enter the early coleoptilar stage with a scutellum structure and partial shoot or root meristems (Figures 1J,M). At 30 DAP, the *dek47-1* embryo remains at the coleoptilar stage with abnormal cell differentiation (Figures 1E,F). The endosperm development in *dek47-1* was delayed as well, and the inhibition of endosperm development in *dek47-1* appeared throughout the whole development process, which is difficult to pinpoint to a specific stage. These results indicate that the embryo development is arrested at the coleoptilar stage and the endosperm development is delayed in *dek47-1*.

**Figure 2:**
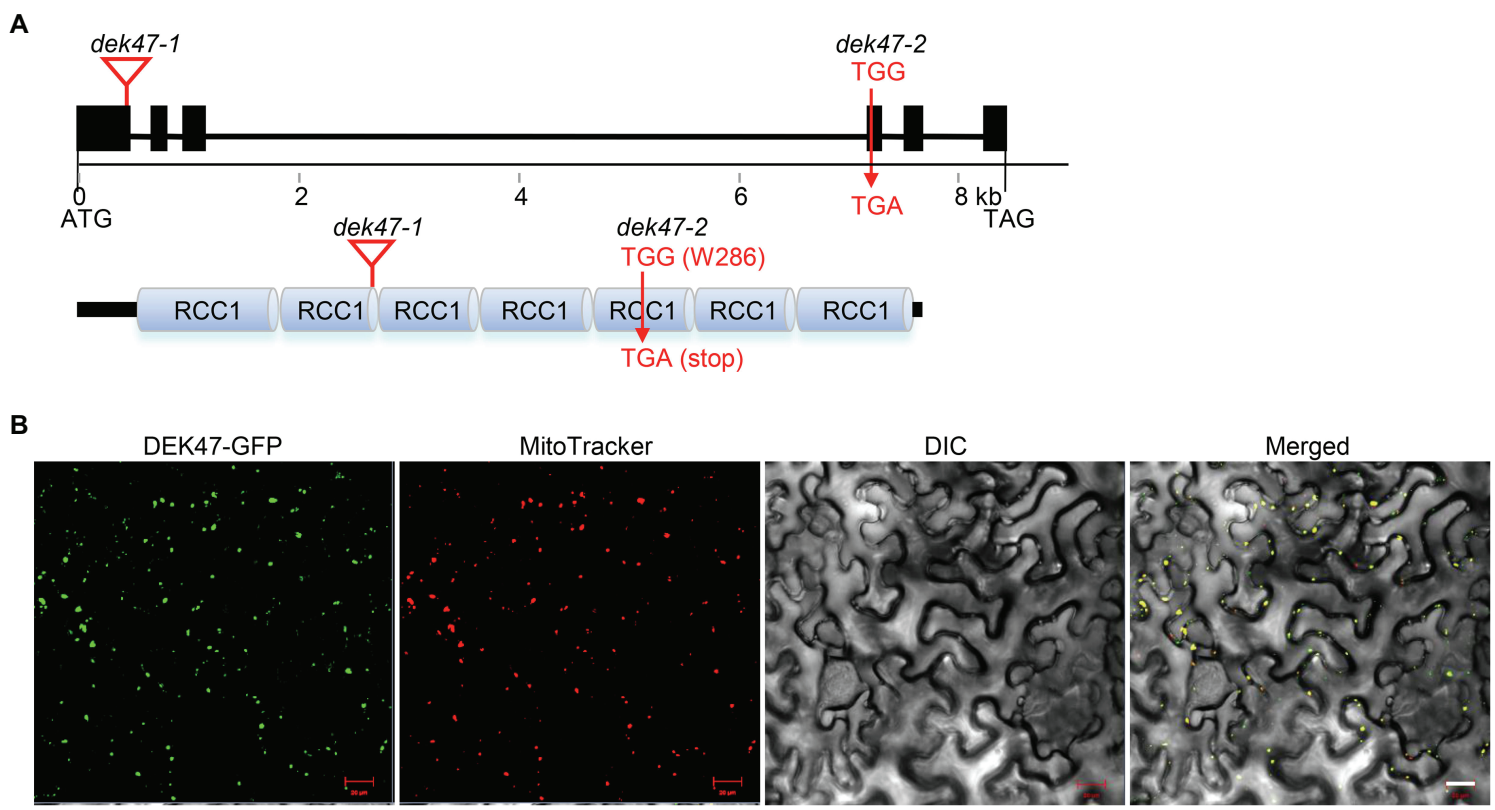
*Dek47* encodes a mitochondrion-targeted RCC1-domain protein. **(A)** Genomic structure of *Dek47* and the motifs of the encoded protein. The top panel represents the gene structure of *Dek47* (blocks, exons; lines, introns) and the below panel represents the protein structures of DEK47. *dek47-1*: locations of *Mu* insertion are marked with triangle. *dek47-2*: the TGG (W286) codon is mutated to TGA (stop). **(B)** The subcellular localization of DEK47 in tobacco leaves. MitoTracker Red was used as a mitochondrial marker. Scale bars = 20 μm.

To determine whether the *dek* phenotype is indeed caused by mutation in *GRMZM2G114748*, we performed linkage analysis. The presence of the *Mu* insertion was verified by PCR with the *Mu* specific primer *Mu*-TIR8- and *GRMZM2G114748*-specific primers, and the homozygote was determined by a lack of wild-type *GRMZM2G114748* and a presence of the *Mu* insertion. The results showed that the *Mu* insertion in *GRMZM2G114748* is tightly linked to the *dek47* phenotype in a population of 48 plants (Supplementary Figure S1). Sequencing result confirmed that the *Dek47-1* mutant carries a *Mu* insertion in *GRMZM2G114748* at 439 bp downstream from the translation start codon ATG (Figure 2A). To genetically confirm that *GRMZM2G114748* is the causal gene, an independent allele was isolated from the ethyl methane sulfonate (EMS)-mutagenized B73 population (Lu et al., 2018), designated *dek47-2*. *dek47-2* contains an EMS-induced G-to-A mutation at +858 bp downstream of the ATG start codon, causing an alteration from TGG (Trp/W) to TGA (stop codon). This mutation terminates the GRMZM2G114748 protein at the 286th amino acid residue, causing a truncation of 146 amino acid residues (Figure 2A). Similar to *dek47-1*, the selfed *dek47-2/+* heterozygotes segregated a quarter of *dek* kernels with a phenotype similar to *dek47-1* (Supplementary Figure S2A). The crossed ears between *dek47-1/+* and *dek47-2/+* heterozygotes produced ~25% *dek* kernels (Supplementary Figure S2B). Thus, we conclude that *GRMZM2G114748* is the causal gene for the *dek47* phenotype, hereafter referred to as *Dek47*.

### *Dek47* Is a Constitutive Gene

*Dek47* contains six exons and five introns, encoding a 45.44-kDa protein with 432 amino acid residues (Figure 2A). Protein domain analysis showed that DEK47 contains seven tandem RCC1 repeats using Prosite^2^ (Figure 2A). Based on the classification by Hadjebi et al. (2008), DEK47 is a typical RCC1 subgroup protein with a character that the RCC1 repeats make up almost the entire protein. RT-PCR analyses showed that no wild-type *Dek47* transcript can be detected in *dek47-1*, indicating that *Dek47* is not expressed in *dek47-1* (Supplementary Figure S3A), suggesting that *dek47-1* is probably a null mutation. Quantitative real-time PCR (qRT-PCR) results showed that the transcript of *Dek47* was detected in all tested tissues with high levels of expression in shoots, leaves, and silks, and low in roots, flowers, and kernels (Supplementary Figure S3B). These data indicate that *Dek47* is a constitutive gene that may play an important role throughout maize growth and development, rather than a seed specific gene.

### DEK47 Localizes in Mitochondria

DEK47 is predicted to have a putative mitochondrial localization signal by TargetP.^3^ To determine its subcellular localization, the full-length *Dek47* was fused to the N-terminus of GFP and the DEK47-GFP fusion was placed under the control of the CaMV 35S promoter in pGWB5. The fusion was transiently expressed in tobacco leaf epidermal cells *via* the *Agrobacterium*-mediated transformation. Strong green fluorescence signals were detected in mitochondria, which were merged with the red fluorescence signals of mitochondria stained by the MitoTracker (Figure 2B), indicating that DEK47 is localized in mitochondria.

### Loss-of-Function of DEK47 Affects Mitochondrial Respiratory Activity

To evaluate whether the mutation of DEK47 affects the mitochondrial respiration, three mitochondrial respiratory rates were measured, the total respiratory (V_t_), cytochrome respiratory capacity (V_cyt_), and alternative respiratory capacity (V_alt_). Compared with WT, V_t_ was sharply decreased to a low level in *dek47-1* (Table 1). The ratio of V_cyt_/V_t_ was markedly decreased and V_alt_/V_t_ strikingly increased in *dek47-1*, implying that the cytochrome respiration pathway is impaired and the alternative respiratory pathway (AOX) is activated as a result of the *Dek47* mutation.

**Table 1:** Alteration of the respiration rate of WT and *dek47-1.*

	Respiration rate (nmol O_2_ min^−1^ g^−1^ fresh weight)				
	V_t_	V_alt_	V_cyt_	V_alt_/V_t_(%)	V_cyt_/V_t_(%)
WT	829.88 ± 70.25	196.46 ± 20.09	700.63 ± 67.55	16.69	84.32
*dek47-1*	102.56 ± 14.29^***^	56.23 ± 9.56^*^	48.60 ± 1.40^***^	62.20^*^	55.68^**^

Mitochondrial total respiration rate (V_t_), the capacity of the cytochrome pathway (V_cyt_), and the alternative pathway (V_alt_) were indicated by O_2_ consumption of the fresh maize kernels at 11 DAP. Data are mean values ± SEs from three independent biological samples. Asterisks indicate significant differences between means calculated with Student’s *t*-test.

* *p < 0.05;*

** *p < 0.01;*

*** *p < 0.001.*

**Figure 3:**
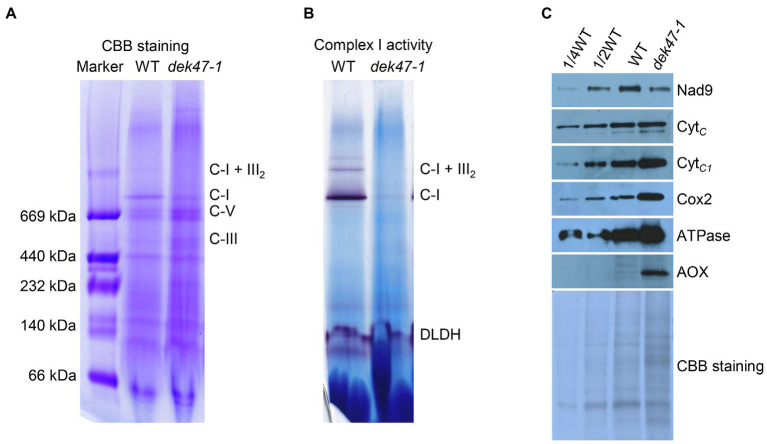
Blue native-PAGE analyses of mitochondrial complexes. **(A)** Assembly of mitochondrial complex I. Mitochondrial complexes of the embryo and endosperm of maize kernels were subjected to a 3–12.5% BN-PAGE. The BN gels were stained with Coomassie brilliant blue (CBB). The position of respiratory complexes is indicated. C-I, complex I; C-III, complex III; C-I+III_2_, supercomplex I +III_2_; and C-V, complex V. **(B)** Detection of NADH dehydrogenase activity of complex I. Dihydrolipoamide dehydrogenase (DLDH) was used as a loading control. **(C)** Analysis of mitochondrial proteins abundance. Western blot analysis with antibodies against Nad9, Cyt*_C_*, Cyt*_C1_*, Cox2, ATPase, and AOX. CBB-stained gel was used as reference of loading quantity.

To determine whether the AOX is enhanced in *dek47-1*, we detected the protein abundance of AOX *via* Western blotting using the specific antibody against AOX. The results showed that the abundance of AOX was drastically increased in *dek47-1*, comparing with the nearly undetectable level of AOX protein in the WT (Figure 3C). There are three AOX genes: *AOX1*, *AOX2*, and *AOX3* in maize (Karpova et al., 2002). RT-PCR results showed that all three *AOX* genes are expressed at undetectable levels in WT, whereas the expression of *AOX2* was dramatically increased in the *dek47* alleles (Supplementary Figure S4A). qRT-PCR analysis provided consistent results (Supplementary Figure S4B), indicating that the AOX pathway is enhanced in *dek47*.

### Loss of DEK47 Affects the Assembly and Activity of Complex I

Dysfunction of mitochondrial respiration can be caused by the defective electron transfer that involves mitochondrial complexes I to IV (Acin-Perez et al., 2008; Millar et al., 2011; Lobo-Jarne and Ugalde, 2018). To determine whether the mitochondrial complex is defective, we employed the BN-PAGE gel analysis and analyzed the complexes by using crude mitochondria isolated from the WT and *dek47-1*. The CBB staining showed that the complex I was markedly reduced, whereas the complexes III and V were increased in *dek47-1* (Figure 3A). In-gel NBT-NADH activity staining results showed that the NADH dehydrogenase activity of complex I was dramatically reduced in the *dek47-1* mutant as the activity of the super CI+CIII_2_ complex was nearly undetectable and CI a fraction of the WT activity (Figure 3B). These data indicate that the mutation of *Dek47* affects the assembly and activity of complex I.

To further assess the effects of the loss of *Dek47* function on mitochondrial complexes, Western blot was performed to detect the abundance of the components of the mitochondrial complexes using antibodies against Nad9 (complex I), Cyt*_C_* (cytochrome *c*), Cyt*_C1_* (complex III), Cox2 (complex IV), and ATPase (complex V). As shown in Figure 3C, the level of Cyt*_C_* showed no difference between WT and *dek47-1*, the level of Nad9 was slightly decreased in *dek47-1*, whereas the levels of Cyt*_C1_*, Cox2, and ATPase were increased in *dek47-1*, suggesting that the deficiency of complex I may promote an accumulation of complexes III, IV, and V in response.

### DEK47 Is Required for the Intron Splicing of *Nad2* Transcript in Mitochondria

**Figure 4:**
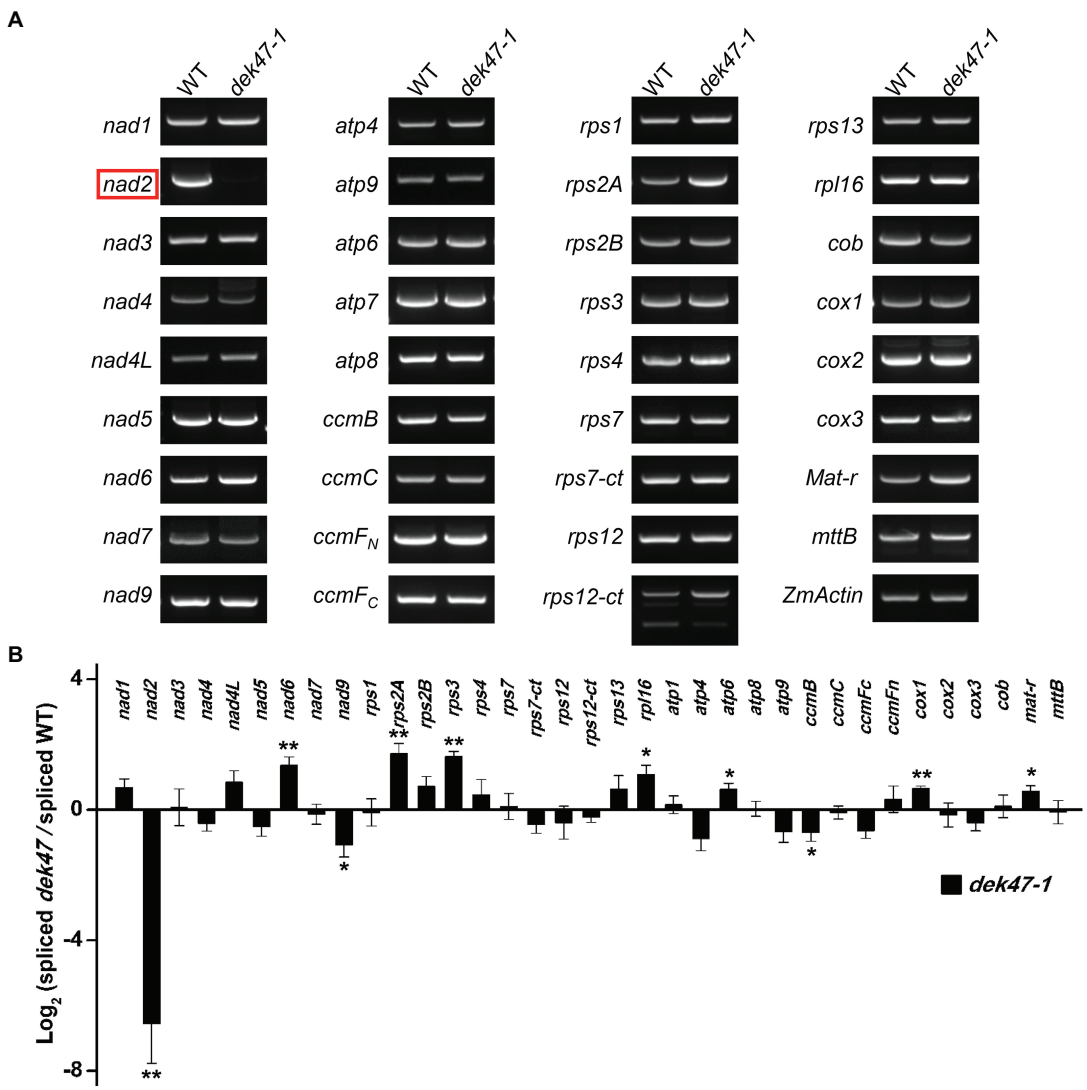
The *Dek47* mutant is deficient for mitochondrial *nad2* mature transcript. **(A)** RT-PCR analysis of transcript levels of 35 mitochondrion-encoded genes in WT and *dek47-1*. RNA was isolated from the same ear segregating for WT and *dek47-1*. Normalization was performed against *ZmActin*. **(B)** Quantitative RT-PCR analysis of 35 mitochondrion-encoded transcripts in WT and *dek47-1*. RNA samples were normalized against *ZmActin*. Values represent the mean and standard deviation of three biological replicates, ±SD. The comparison groups of the Student’s *t*-test are wild type and *dek47* mutant. Asterisks indicate significant differences between means calculated with Student’s *t*-test. ^*^p < 0.05; ^**^p < 0.01.

The deficiency of mitochondrial complex I can be caused by a deficiency of the complex I components or the assembly factors (Klodmann et al., 2010). To investigate whether the mitochondrial complex components are affected, we analyzed the transcripts of 35 mitochondrion-encoded genes by RT-PCR and qRT-PCR analysis in the WT and *dek47-1* kernels at 12 DAP. Results showed that the expression levels of the 34 mitochondrial genes were comparable between the WT and *dek47-1*, except for *nad2* which was barely detectable in the mutant (Figures 4A,B).

**Figure 5:**
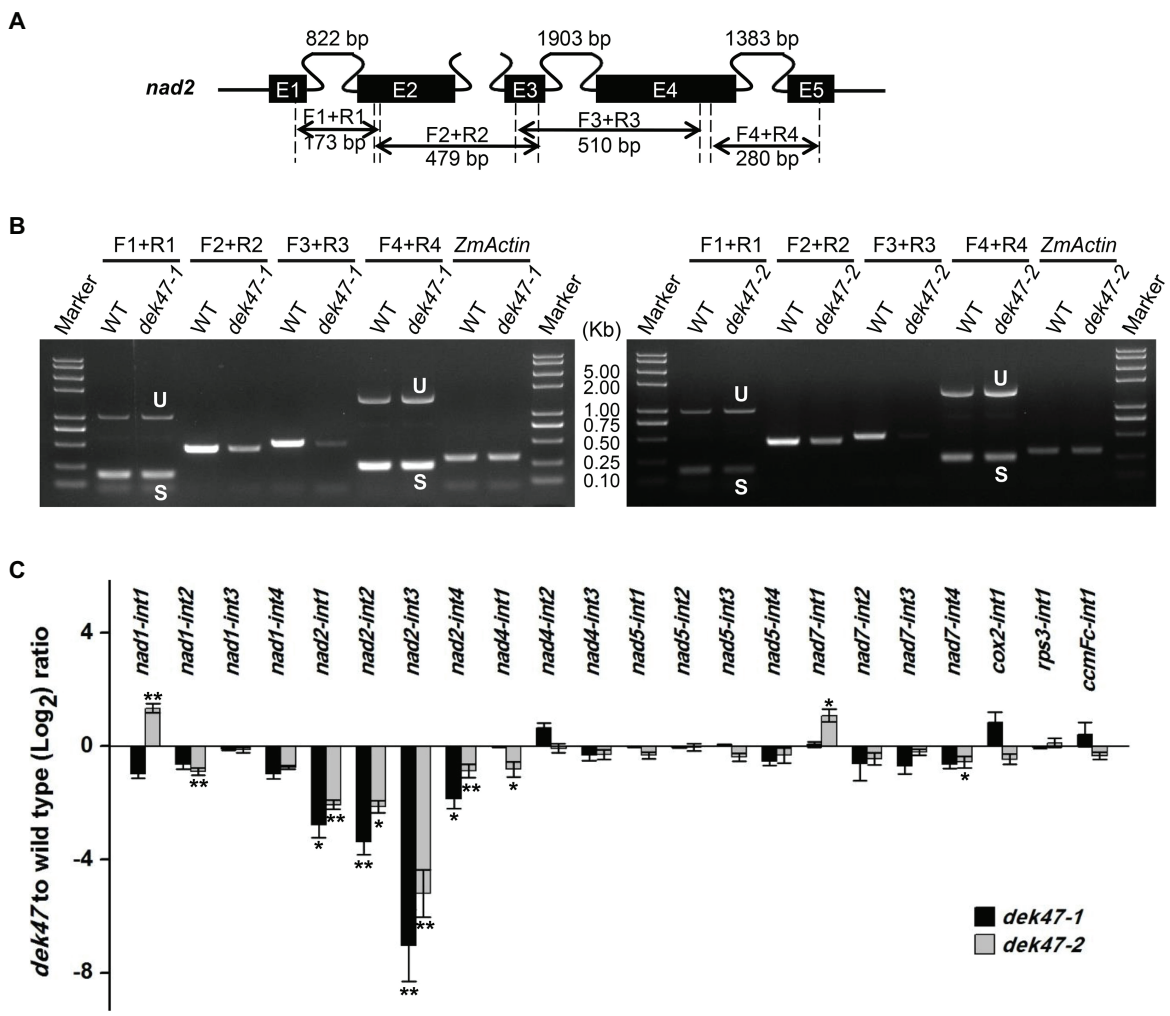
The intron splicing of *nad2* transcript is impaired in *dek47*. **(A)** Schematic representation of *nad2* gene. Intron 1, intron 3, and intron 4 of *nad2* are *cis*-splicing introns. The expected amplification products and primers using are indicated. F and R present the primers used to detect splicing events in RT-PCR analysis. E1-E5, exon1-exon5. **(B)** RT-PCR analysis of the splicing of *nad2* introns in WT and *dek47*. Amplifications are marked as in **(A)**. Normalization was performed against *ZmActin*. S and U indicate the spliced and unspliced PCR products, respectively. **(C)** Quantitative RT-PCR analysis of 22 introns splicing efficiency of mitochondrial genes in WT and *dek47*. RNA samples were normalized against *ZmActin*. Values represent the mean and standard deviation of three biological replicates, ±SD. The comparison groups of the Student’s *t*-test are wild type and *dek47* mutant. Asterisks indicate significant differences between means calculated with Student’s *t*-test. ^*^p < 0.05; ^**^p < 0.01.

The maize mitochondrial *nad2* gene contains 5 exons and 4 introns. Primers were designed to anchor on the exons across each intron (Figure 5A). RT-PCR results indicated that the splicing of all four *nad2* introns was reduced in *dek47-1* with a substantial decrease in intron 3 (Figure 5B), suggesting that *Dek47* is required for splicing of all four introns of *nad2* transcript. To evaluate the impact on other introns, we compared the splicing efficiency of the mitochondrial 22 group II introns between the WT and *dek47* mutants by qRT-PCR (Figure 5C). Results showed that the splicing efficiency of the four *nad2* introns was decreased in *dek47-1* compared with that in WT (Figure 5C), whereas the splicing of other introns appeared normal. These results are consistent with the RT-PCR results, suggesting that *Dek47* is indeed required for the splicing of the four introns of *nad2* transcript.

**Figure 6:**
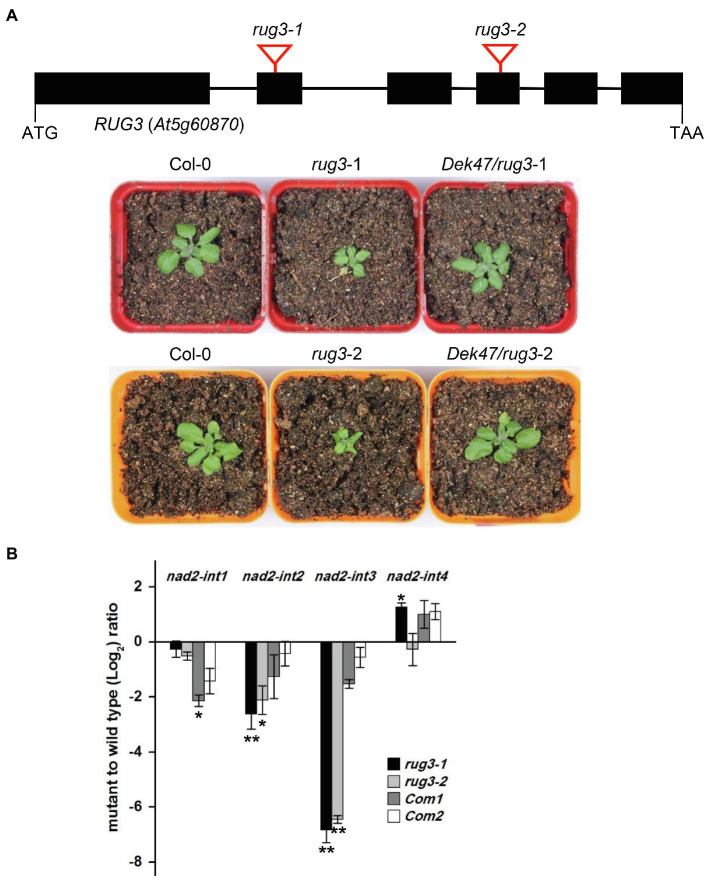
DEK47 can rescue the Arabidopsis *rug3* mutant phenotypes. **(A)** Phenotype comparison of Col-0, *rug3*, *Dek47/rug3-1* (*Com1*), and *Dek47/rug3-2* (*Com2*). **(B)** Quantitative RT-PCR analysis of intron splicing efficiency of *nad2* transcript in WT, *rug3*, *Com1*, and *Com2*. RNA samples were normalized against Actin gene *ACT2 (AT3G18780)*. Values represent the mean and standard deviation of three biological replicates, ±SD. The comparison groups of the Student’s *t*-test are between *rug3* mutant and wild type, and between complemented line and wild type, respectively. Asterisks indicate significant differences between means calculated with Student’s *t*-test. ^*^p < 0.05; ^**^p < 0.01.

A previous study showed that RUG3 protein functions in the splicing of *nad2* introns 2 and intron 3 in Arabidopsis mitochondria (Kuhn et al., 2011). BLAST analysis indicates that DEK47 shares a 63% sequence identity with AT5G60870 (RUG3) from Arabidopsis (Supplementary Figure S5). To address the functional relationship between DEK47 and RUG3, *Dek47* was expressed in the *rug3-1* and *rug3-2* homozygotes to test whether DEK47 could rescue the Arabidopsis *rug3* mutant phenotype. Two Arabidopsis T-DNA insertion lines *rug3-1* (SALK_092071) and *rug3-2* (GABI_466G01) were obtained from the Arabidopsis Biological Resource Center (Figure 6A). The two alleles of *rug3* are defective in the intron splicing of *nad2* and show developmentally delayed phenotypes (Kuhn et al., 2011). The genotypes of *rug3* mutants and complemented lines *Dek47/rug3-1* (*Com1*) and *Dek47/rug3-2* (*Com2*) were confirmed by PCR analysis (Supplementary Figure S6). The *rug3-1* and *rug3-2* homozygous plants that carried the *Dek47* transgene in *Com1* and *Com2* exhibited normal growth and development comparing with the wild-type (Figure 6A). qRT-PCR results revealed that the splicing efficiency of *nad2* introns was largely restored in the complemented plants *Com1* and *Com2* (Figure 6B). These data suggest that DEK47 can complement RUG3 in Arabidopsis and is likely the ortholog of RUG3 in maize.

### DEK47 May Not Physically Interact With Related Splicing Factors

**Figure 7:**
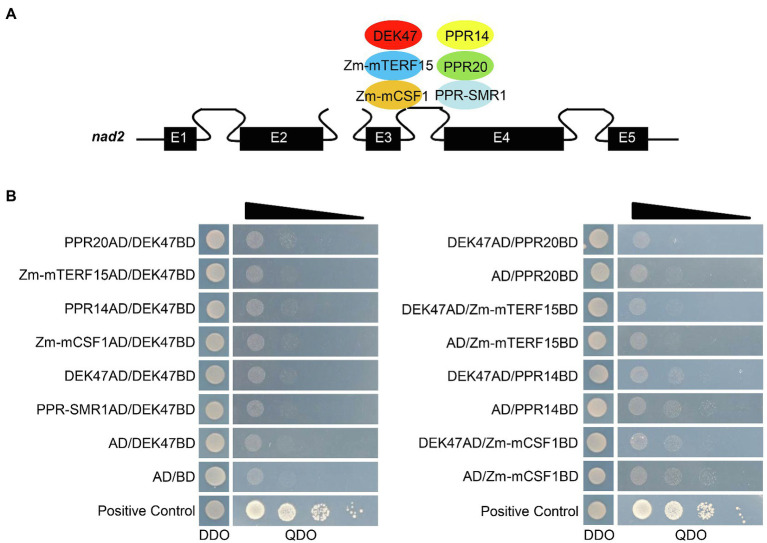
Interaction assay of DEK47 and the related splicing factors. **(A)** Schematic representation of *nad2* transcript and the related splicing factors of *nad2* intron 3. E1-E5, exon1-exon5. **(B)** Yeast two-hybrid assay of DEK47 and the related splicing factors. These constructs were co-transfected into Y2H Gold strain and spotted onto DDO (SD/−Leu/−Trp) medium and QDO (SD/−Ade/-His/−Leu/−Trp) medium for 4 days at 30°C.

Previous studies reported that the splicing factors Zm-mTERF15, Zm-mCSF1, PPR14, PPR20, and PPR-SMR1 are involved in the splicing of mitochondrial *nad2* intron 3 in maize (Chen et al., 2019; Wang et al., 2020; Yang et al., 2020). Thus, we tested whether DEK47 can physically interact with these proteins by Y2H (Figure 7A). Results showed that co-transformants between DEK47 and these splicing factors did not grow on QDO (SD/−Ade/-His/−Leu/−Trp) medium except the positive control (Figure 7B), suggesting that DEK47 may not directly interact with these splicing factors in the Y2H system. To independently test that result, the luciferase complementation imaging (LCI) assay was performed. DEK47, Zm-mTERF15, Zm-mCSF1, PPR14, PPR20, and PPR-SMR1 were fused to the NLUC and CLUC, respectively. Co-expression of the combination of ZmMORF8-NLUC and ZmMORF1-CLUC was used as a positive control (Liu et al., 2020b). No luciferase activity was detected in the tobacco leaves co-expressing DEK47 and the above splicing factors (Supplementary Figure S7). These results indicate that DEK47 may not physically interact with the related splicing factors.

Co-transformants of DEK47AD/DEK47BD did not grow on QDO medium (Figure 7B), and co-transfection of DEK47-NLUC and DEK47-CLUC failed to produce a visible luciferase activity signal (Supplementary Figure S7), indicating that DEK47 cannot form a dimer or multimer by itself in the Y2H and LCI system.

## Discussion

### DEK47 is Essential for the Splicing of *Nad2* Introns, Complex I Biogenesis, and Seed Development in Maize

RCC1 motif is defined as a conserved domain consisted of 51–68 amino acid residues to fold into a bladed beta-propeller in many eukaryotes (Renault et al., 1998). The human RCC1 protein is involved in mitosis, nucleo-cytoplasmic transport, and nuclear-envelope assembly as a signal for chromosome position (Hetzer et al., 2002). In *A. thaliana*, four RCC1-domain proteins (UVR8, RUG3, TCF1, and SAB1) have been characterized (Brown et al., 2005; Kuhn et al., 2011; Ji et al., 2015, 2019). In maize, 31 putative RCC1-like proteins are identified; however, none of which have been characterized at the molecular level. In this study, we revealed the function of a RCC1 family protein DEK47 and its critical role in kernel development in maize.

Genetic evidence demonstrates that DEK47 loss of function arrests both embryo genesis and endosperm development, giving rise to a *dek* phenotype in maize (Figure 1). *dek47-1* contains a *Mu* insertion in the second RCC1 motif, and *dek47-2* contains a point mutation causing a translation stop codon in the fifth RCC1 motif. Both mutants exhibited the *dek* phenotype, and the crosses between the two alleles produced the *dek* phenotype as well. Molecular analyses indicated that the absence of DEK47 reduces the splicing efficiency of the four introns of *nad2*, especially the splicing of *nad2* intron 3 in *dek47* alleles (Figure 5C), suggesting that DEK47 is required for the splicing of four *nad2* introns in maize mitochondria.

Nad2 is a central subunit of complex I on the mitochondrial membrane and essential to the assembly of complex I (Klodmann et al., 2010; Ligas et al., 2019). Previous studies have reported that the absence of Nad2 results in disassembly and severely reduced activity of complex I, leading to the delayed growth and abnormal seed development. For instance, loss-of-function of *Emp16*, *Emp10*, *Emp12*, *Dek37*, and *PPR20* exhibited severely disassembly and impaired activity of mitochondrial complex I and arrested embryo and endosperm development in maize, leading to embryo lethality caused by the splicing deficiency of *nad2* transcript (Xiu et al., 2016; Cai et al., 2017; Dai et al., 2018; Sun et al., 2019; Yang et al., 2020). Similar cases were also occurred in the *dek47* mutants, implying the vital roles of DEK47 in mitochondrial complex I biogenesis and seed development in maize.

The OXPHOS pathway is the major respiration pathway in plant cells where electrons from NADH and FADH_2_ are transferred *via* complex I to IV to O_2_ to produce H_2_O. Along with this electron transfer, protons are pumped out to the inner and outer membrane space. Proton flow from the high concentration space to the low concentration matrix drives mitochondrial complex V (ATPase) to synthesize ATP. Complex I is the entry point of the electron transfer chain. The mutation of DEK47 affects the splicing of the *nad2* introns, leading to a deficiency of Nad2 protein and a disassembly of Complex I (Figure 3A). As such, it blocks the OXPHOS pathway and stimulates the expression of the AOX pathway. The deficiency of Nad2 blocks the respiration pathway is found in several mutants, such as *emp16* (Xiu et al., 2016), *emp8*, and *emp12* (Sun et al., 2018, 2019).

### Evolutionary Conservation and Divergence Between DEK47 and RUG3 on *Nad2* Introns Splicing

Alignment analysis indicates that DEK47 protein shares a 63% identity with RUG3 in Arabidopsis (Supplementary Figure S5), suggesting that these two are likely orthologs. At the molecular level, both DEK47 and RUG3 are required for the splicing of mitochondrial *nad2* introns 2 and 3 (Kuhn et al., 2011), suggesting that DEK47 and RUG3 are functional orthologs. However, DEK47 is also required for the splicing of *nad2* introns 1 and 4, which is not observed in RUG3 (Figure 5), suggesting that DEK47 may have gained additional function in facilitating the splicing of other introns in contrast to RUG3 in Arabidopsis. This functional divergence is common to orthologous pairs between maize and Arabidopsis, such as PPR2263/MEF9 and ZmDEK36/ATDEK36 (Sosso et al., 2012; Wang et al., 2017). PPR2263 functions in the editing of mitochondrial *nad5*-1,550 and *cob*-908 sites in maize, whereas MEF9 only participates in the editing at *nad5*-1550 site in Arabidopsis. Maize DEK36 is only required for mitochondrial RNA editing at multiple sites, whereas AtDEK36 is involved in RNA stabilization except for RNA editing. Thus, functions of orthologous RNA editing factors and splicing factors have diverged to cope with genetic alterations in mitochondria during the evolution of maize and Arabidopsis. In that reasoning, one possible explanation is possible that genetic alteration in the maize *nad2* introns 1 and 4 invokes the recruitment of DEK47, but not in the case of Arabidopsis *nad2* introns 1 and 4 for RUG3, and another one could be the difference in available splicing factors between the two species.

### Splicing of Individual Intron Requires Multiple Splicing Factors

Previous studies reported that different types of splicing factors are required for intron splicing in plant mitochondria, such as maturases, CRM-domain protein, RNA DEAD helicases, PPR proteins, PORR domain family proteins, and RCC1 proteins (Brown et al., 2014). Among these splicing factors, PPR protein PPR14, PPR-SMR1, and CRM-domain protein Zm-mCSF1 interacts with each other to facilitate the splicing of *nad2* intron 3 (Chen et al., 2019; Wang et al., 2020). However, how these factors coordinate to function in intron splicing is not clear. In this study, the splicing efficiency of four *nad2* introns is decreased in *dek47*, whereas the splicing of other mitochondrial introns appears indistinguishable compared with WT (Figure 5C), suggesting that DEK47 is specifically required for the intron splicing of *nad2*. However, RCC1-like proteins have not been shown to possess the RNA-binding activity. Instead, RCC1-domain proteins is shown to directly interact with proteins and lipids to mediate diverse biological processes in animals (Hadjebi et al., 2008). The RCC1-domain proteins and the WD40-repeat proteins share a similar seven-bladed propeller in tertiary structure (Hadjebi et al., 2008). Surface electrostatics of seven-bladed WD structures is highly conserved. The seven-bladed WD beta-propellers have multiple binding partners and are involved in protein–protein interactions (Valeyev et al., 2008). Based on that, it is reasonable to speculate that the 7 RCC1 domains of DEK47 also form seven-bladed beta-propellers to participate in *nad2* intron splicing through interacting with other proteins, such as the PPRs. Previous studies have identified five splicing factors that are involved in the splicing of *nad2* intron 3 in maize, including PPR14, PPR20, PPR-SMR1, Zm-mCSF1, and Zm-mTERF15 (Chen et al., 2019; Wang et al., 2020; Yang et al., 2020). However, our protein interaction analyses showed that DEK47 did not physically interact with these splicing factors in the Y2H analysis and LCI assay (Figure 7, Supplementary Figure S7). It is possible that DEK47 may recruit other unknown factors to participate in the splicing of *nad2* intron 3, or such interaction is too weak or transient to be detected by the Y2H and LCI assays.

### The Importance of Respiratory Complex I to Embryo Lethality in Monocots and Dicots

We observed a marked difference in the dependence on complex I for plant growth and development between Arabidopsis and maize. The deficiency of mitochondrial complex I usually leads to embryo lethality in maize, whereas such mutants can survive and set seeds in Arabidopsis. For example, the loss-of-function of EMP16 and DEK47 affects the assembly of complex I, leading to severely aborted embryo and endosperm development in maize (Xiu et al., 2016). But the mutants of *otp43, ndufs4*, and *slo1* with a complete loss in the assembly and activity of complex I in Arabidopsis can survive and set seeds (de Longevialle et al., 2007; Meyer et al., 2009; Sung et al., 2010). It is not clear why such a difference exists between maize and Arabidopsis. One possibility is that the maize embryogenesis is more sensitive to the OXPHOS than that of Arabidopsis. The loss of respiratory complex I occurred several times in unicellular eukaryotes during evolution. In multicellular plant species, the mitochondrial complex I of European mistletoe (*Viscum album*) is absent and the complex I activity cannot be detected (Maclean et al., 2018; Senkler et al., 2018), implying that complex I is not essential for mitochondrial function in European mistletoe. In plants, the classical OXPHOS electron transport chain (Complexes I, IV, III, and IV) and non-energy conserving alternative electron transport bypasses (alternative oxidase and NAD(P)H dehydrogenases) are present to regulate mitochondrial respiration (Millar et al., 2011). When the function of complex I is impaired, alternative electron transport bypass is enhanced in plants. Consequently, the corresponding Arabidopsis mutants can survive with a low phosphorylation efficiency *via* the adjustment in cellular metabolism and development (Meyer et al., 2009), whereas the maize mutants cannot as observed in *emp8*, *emp10*, and *emp16* (Xiu et al., 2016; Cai et al., 2017; Sun et al., 2018). The molecular basis of this difference invites further investigation.

## Data Availability Statement

The original contributions presented in the study are publicly available. This data can be found here: Sequence data for *Dek47* can be found in GenBank (http://www.ncbi.nlm.nih.gov) under the accession number *GRMZM2G114748*.

## Author Contributions

S-KC, RL, and B-CT designed the experiments, analyzed the data, and wrote the manuscript. S-KC, RL, AS, FS, RS, XW, ZX, and XL performed the experiments. All authors contributed to the article and approved the submitted version.

## Conflict of Interest

The authors declare that the research was conducted in the absence of any commercial or financial relationships that could be construed as a potential conflict of interest.

## Publisher’s Note

All claims expressed in this article are solely those of the authors and do not necessarily represent those of their affiliated organizations, or those of the publisher, the editors and the reviewers. Any product that may be evaluated in this article, or claim that may be made by its manufacturer, is not guaranteed or endorsed by the publisher.

## Footnotes

^1^ http://www.elabcaas.cn/memd/

^2^ https://prosite.expasy.org/

^3^ http://www.cbs.dtu.dk/services/TargetP
