# Supplementary material for: Regulator of Chromosome Condensation 1-Domain Protein DEK47 Functions on the Intron Splicing of Mitochondrial Nad2 and Seed Development in Maize
Source: Front Plant Sci. 2021 Aug 2;12:695249. doi: 10.3389/fpls.2021.695249 (PMC8365749; doi:10.3389/fpls.2021.695249)
Supplement: Supplementary file 1 [file Data_Sheet_1.PDF]

Supplementary Material

Figure. S1:

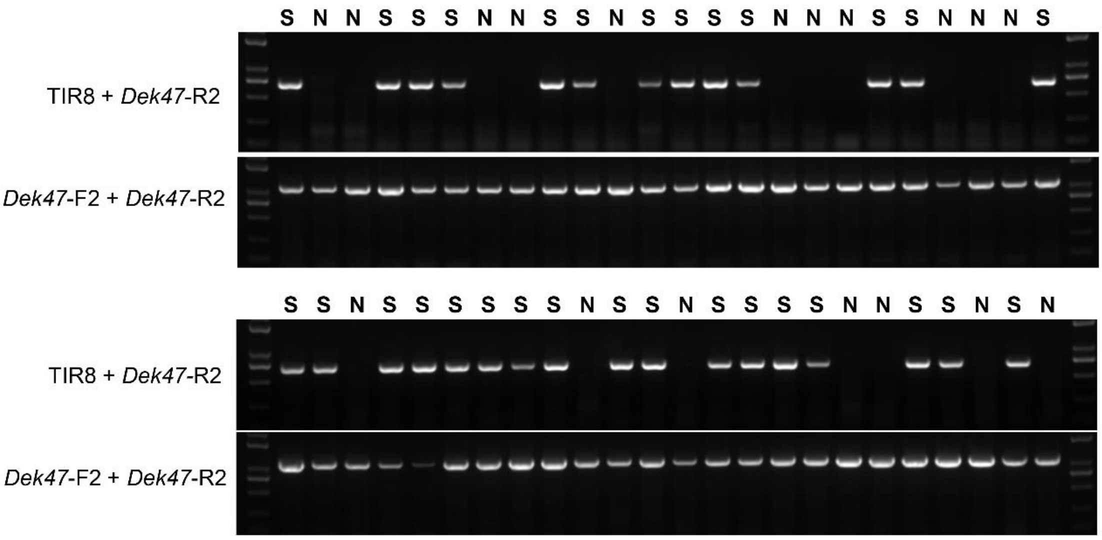

Figure S1. Linkage analysis in an F2 population segregating *dek47-1*.

TIR8 and *Dek47*-R2 are primers used to detect the *Mu* insertion of *Dek47* mutants, *Dek47*-F2+*Dek47*-R2 are the primers used to detect the *Dek47* gene.

N, non-segregating; S, segregating.

Figure S2:

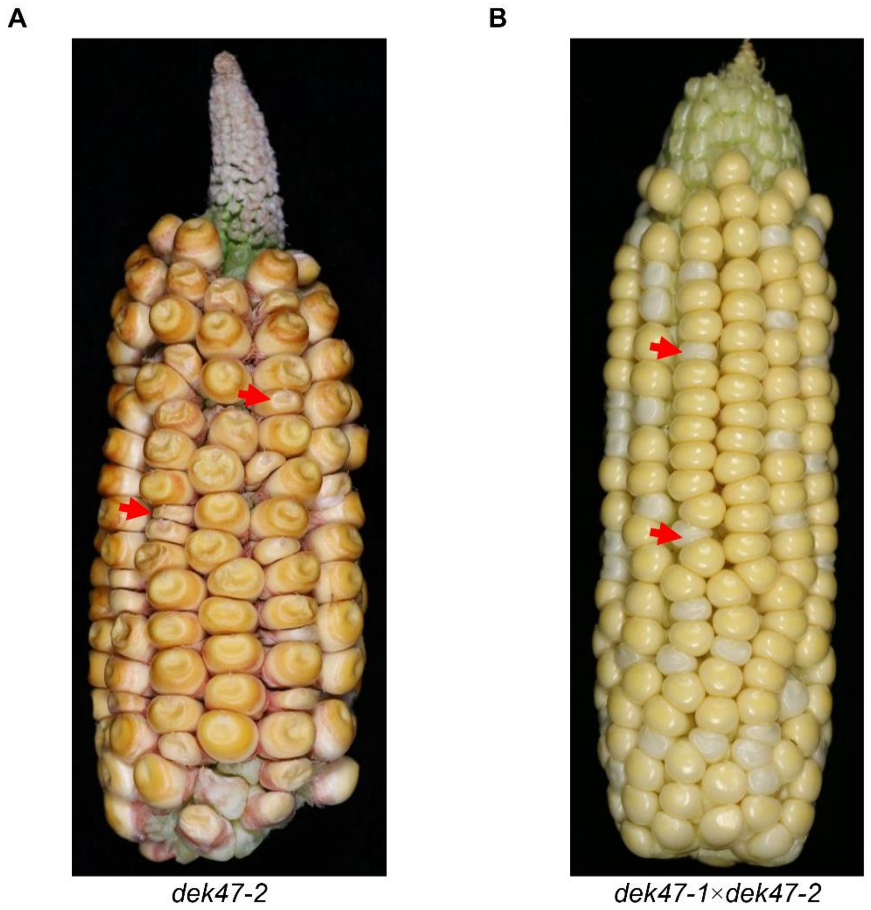

Figure S2. Phenotypes of the *dek47-2* allele and the ear of *dek47-1×dek47-2*.

(A) A segregating ear of *dek47-2*. Arrowhead indicates *dek47-2* kernels.

(B) The phenotype of the ear of heterozygote *dek47-1×dek47-2* cross. Arrows indicate the mutant kernels.

Figure S3:

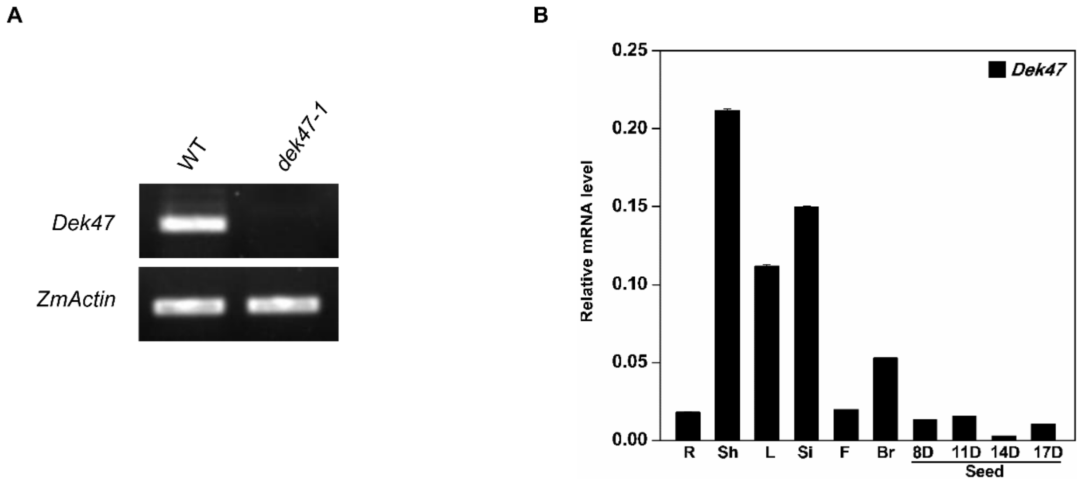

Figure S3. Expression analysis of *Dek47*.

(A) RT-PCR analysis of *Dek47* expression in *dek47-1* and wildtype (WT).

(B) qRT-PCR analysis on the expression of *Dek47* in major tissues and developing seeds in maize. RNAs were normalized against maize *Actin* gene (*GRMZM2G126010*). R: Root; Sh: Shoot; L: Leaf; Si: Silk; Fl: Flower; Br: Bract. D: days after pollination (DAP). Values represent the mean and standard deviation of three biological replicates, ±SD.

Figure S4:

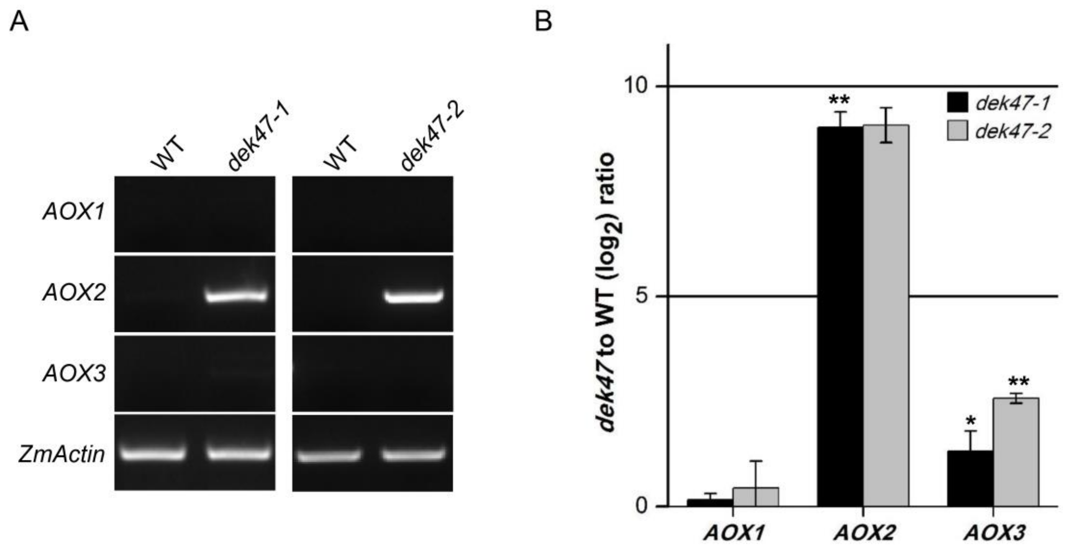

Figure S4. Transcription profiling of *AOX* genes in WT and *dek47*.

(A) RT-PCR analyses of *AOX* genes in WT and *dek47*.

(B) qRT-PCR analyses of *AOX* genes in WT and *dek47*. RNAs were normalized against *Actin* gene (*GRMZM2G126010*). Values represent the mean and standard deviation of three biological replicates, ±SD. The comparison groups of the Student's t-test analyses are wild type and *dek47* mutant. Asterisks indicate significant differences between means calculated with Student's t-test. *P < 0.05; **P < 0.01.

Figure S5:

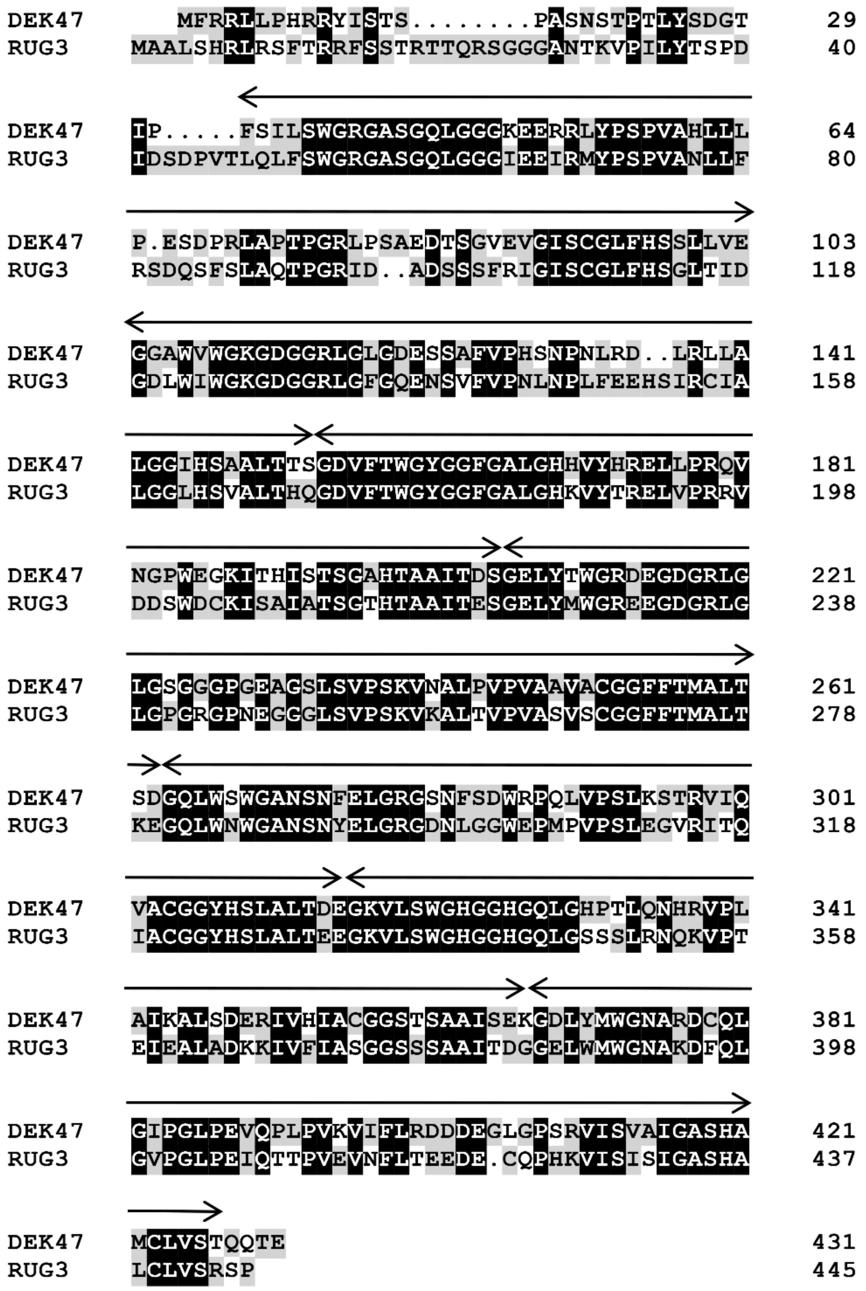

Figure S5. BLAST analysis of maize DEK47 with Arabidopsis RUG3.

Arrowhead indicates each RCC1 motif.

Figure S6:

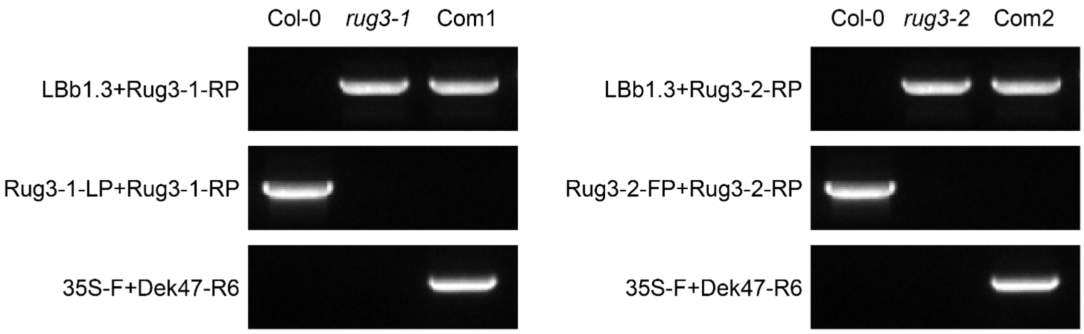

Figure S6. Genotype of *rug3* mutants and the complementation lines.

Figure S7:

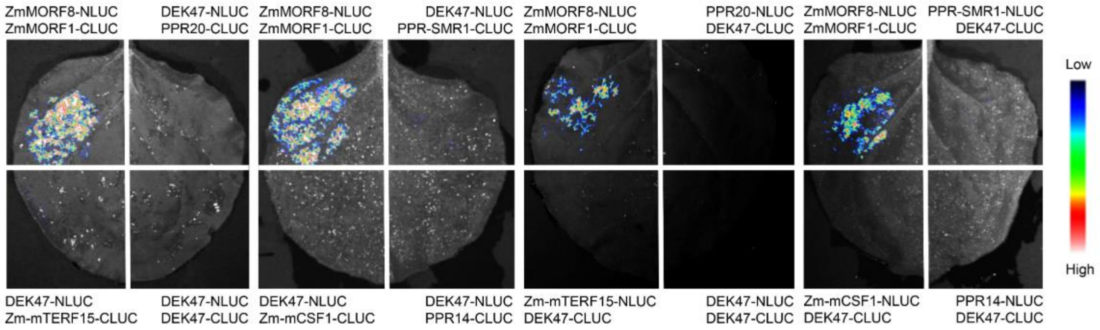

Figure S7. DEK47 does not interact with related splicing factors in LCI assay.

Interaction analysis of DEK47 and related splicing factors (Zm-mTERF15, Zm-mCSF1, PPR14, PPR20, and PPR-SMR1) by luciferase complementation imaging assay in *N. benthamiana*. The combination of ZmMORF8-NLUC and ZmMORF1-CLUC was co-expressed in tobacco leaves as positive control. The intensity of the fluorescent signals represents their interaction activities.
