# Supplementary material for: Regulator of Chromosome Condensation 1-Domain Protein DEK47 Functions on the Intron Splicing of Mitochondrial Nad2 and Seed Development in Maize
Source: Front Plant Sci. 2021 Aug 2;12:695249. doi: 10.3389/fpls.2021.695249 (PMC8365749; doi:10.3389/fpls.2021.695249)
Supplement: Supplementary file 2 [file Data_Sheet_2.PDF]

Supplementary Table S1

Primers used in this study.

Primer name	Primer sequences (5'-3')	Application
Dek47-F1	CACCATGTTCCGCCGCCTCCTCCC	*Dek47* cloning into pEntry vector
Dek47-R1	CTTCTCGGTTTGTTGCGTGGAGACC	
Dek47-F2	TTCGTGTCCTCTGGTCTCTCGC	Genotype *dek47-1* mutant
Dek47-R2	CATTCACCTTGGAAGGAACGCTG	
Dek47-F3	TAGAGTTGAGACGGGGGGGGG	Genotype *dek47-2* mutant
Dek47-R3	TGATTTTGAAGGGTGGGATGCC	
Dek47-F4	CTTCCACTCCTCGCTCCTCGT	RT-PCR analysis of *Dek47* gene
Dek47-R4	ACTCCCAAGCCCCAGCCTAC	
Dek47-F5	GCCCCACTCTAACCCCAATCT	qRT-PCR analysis of *Dek47* gene
Dek47-R5	CTTGGCAAAAGCTCCCTATGG	
Dek47-YTH-F	GAATTCTTCTCTATCCTCTCGTGGGGC	Yeast two-hybrid analysis
Dek47-YTH-R	GGATCCCTTCTCGGTTTGTTGCGTGGA	
Dek47-cLUC-F	GAGAACACGGGGGACGAGCTCATGTTCCGCCGCCTCCTCCC	Luciferase Complementation Imaging Assay
Dek47-cLUC-R	TACATAACCGGACATGAGCTCCTTCTCGGTTTGTTGCGTGGAGACC	
Dek47-nLUC-F	CGAGCTCGGTACCCGGGATCCATGTTCCGCCGCCTCCTCCC	
Dek47-nLUC-R	CGCGTACGAGATCTGGTCGACCTTCTCGGTTTGTTGCGTGGAGACC	
PPR14-cLUC-F	GAGAACACGGGGGACGAGCTCATGCGTCGCTACTGCCACGT	
PPR14-cLUC-R	TACATAACCGGACATGAGCTCTTCAAACAGTGTTTGAAATTTC	
PPR14-nLUC-F	CGAGCTCGGTACCCGGGATCCATGCGTCGCTACTGCCACGT	
PPR14-nLUC-R	CGCGTACGAGATCTGGTCGACTTCAAACAGTGTTTGAAATTTC	
PPR20-cLUC-F	GAGAACACGGGGGACGAGCTCATGGCGCTCGCCACGCTGC	
PPR20-cLUC-R	TACATAACCGGACATGAGCTCGGAAGATGTCAAGTTTTTAGAAAC	
PPR20-nLUC-F	CGAGCTCGGTACCCGGGATCCATGGCGCTCGCCACGCTGC	
PPR20-nLUC-R	CGCGTACGAGATCTGGTCGACGGAAGATGTCAAGTTTTTAGAAAC	
PPR-SMR1-cLUC-F	GAGAACACGGGGGACGAGCTCATGCTGCTCCGCGTTGGC	
PPR-SMR1-cLUC-R	TACATAACCGGACATGAGCTCCCTAGGCATGCCAAGGGATCT	
PPR-SMR1-nLUC-F	CGAGCTCGGTACCCGGGATCCATGCTGCTCCGCGTTGGC	
PPR-SMR1-nLUC-R	CGCGTACGAGATCTGGTCGACCCTAGGCATGCCAAGGGATCT	
Zm-mCSF1-cLUC-F	GAGAACACGGGGGACGAGCTCATGCTCACCCTCCCCGGTAC	
Zm-mCSF1-cLUC-R	TACATAACCGGACATGAGCTCAATTACTTTTGTAATTTGGCAC	
Zm-mCSF1-nLUC-F	CGAGCTCGGTACCCGGGATCCATGCTCACCCTCCCCGGTAC	
Zm-mCSF1-nLUC-R	CGCGTACGAGATCTGGTCGACAATTACTTTTGTAATTTGGCAC	
Zm-mTERF15-cLUC-F	GAGAACACGGGGGACGAGCTCATGGCCACCACCACATCTCCA	
Zm-mTERF15-cLUC-R	TACATAACCGGACATGAGCTCTTTCAGTGATCCCACGAGAGAC	

Zm-mTERF15-nLUC-F	CGAGCTCGGTACCCGGGATCCATGGCCACCACCACATCTCCA	
Zm-mTERF15-nLUC-R	CGCGTACGAGATCTGGTCGACTTTCAGTGATCCCACGAGAGAC	
Zmnad2-F1	AGTAATGTGGGTTGGCTTGG	RT-PCR analysis of *Zmnad2* transcript
Zmnad2-R1	GAAATGGTACCAGCCGTACTT	
Zmnad2-F2	TTTTTTTAGGAGGGACAATTT	
Zmnad2-R2	GGAACTGCAGTAATCTTGAATAGGG	
Zmnad2-F3	TCTACTGGAGCTACCCACTTCGA	
Zmnad2-R3	GGTTTGCCGTAATGCTGGA	
Zmnad2-F4	TCTCATACGCAGGAATACCCCC	
Zmnad2-R4	AAGATCGAACAAGGGAGAGGGA	
Zmnad2-int1F	AGTAATGTGGGTTGGCTTGG	Test *Zmnad2* introns splicing efficiency
Zmnad2-int1R	GAAATGGTACCAGCCGTACTT	
Zmnad2-exonF1	GCGGTTTCCCCAGAGATCTTTC	
Zmnad2-intronR1	TACGATTAGCCAGCCTTGCGGC	
Zmnad2-int2F	TCGCAGCATCAAAAAGAAAG	
Zmnad2-int2R	GATCGAAGTGGGTAGCTCCA	
Zmnad2-exonF2	TGATCTTAGGTGCATTTCCCTCTG	
Zmnad2-intronR2	ATCGGTAGTAGTCCGGTCGCAC	
Zmnad2-int3F	ACCGGATACGAAATCACTGG	
Zmnad2-int3R	GCGCAATAGAAAGGAATGCT	
Zmnad2-exonF3	TCTACTGGAGCTACCCACTTCGA	
Zmnad2-intronR3	AGCGGTACCACCCATCCTACC	
Zmnad2-int4F	GGTTGTGGGGCTTACTTCCT	
Zmnad2-int4R	CGACTTGTCACGATCCATTG	
Zmnad2-exonF4	TTCCAGCATTACGGCAAACC	
Zmnad2-intronR4	TACTCATGGCAACCTTCCGGC	
qAtnad2-e1-F	GGATCCTCCCACACATGTTC	Test *Atnad2* introns splicing efficiency
qAtnad2-i1-F	CCCATTCCTAACCAGTGGAG	
qAtnad2-e2-R	GCGAGCAGAAGCAAGGTTAT	
qAtnad2-e2-F	AATATTTGATCTTAGGTGCATTTTC	
qAtnad2-i2-F	GTGTAGTGTGGTGGTTGGGC	
qAtnad2-e3-R	AAAGGAACTGCAGTGATCTTGA	
qAtnad2-e3-F	CTATGGGTCTACTGGAGCTACCC	
qAtnad2-i3-F	GGCGAATTTCAAACTTGTGG	
qAtnad2-e4-R	GCGCAATAGAAAGGAATGCT	
qAtnad2-e4-F	TTGGGTTGTGGGGCTTACTTT	
qAtnad2-i4-F	TTTGGAGAGGACTCAGCTGTT	

qAtnad2-e5-R	ACTGAGAACAAAGGAGAGGGG	
AtActin-qRT-F	CTTGCACCAAGCAGCATGAA	
AtActin-qRT-R	CCGATCCAGACACTGTACTTCCTT	
AOX1-F1	CCTATTGGACCGTCAAATTACTGC	RT-PCR/qRT-PCR analysis of *AOX1* gene
AOX1-R1	CACTGTTTCCAGCATCATAGCAC	
AOX2-F1	GACATCTTCTTCCAGAGGCG	RT-PCR analysis of *AOX2* gene
AOX2-R1	TGACTACGTCCTTGAGCGTG	
AOX2-F2	CCAAGACGCTGATGGATAAGGT	qRT-PCR analysis of *AOX2* gene
AOX2-R2	CCACGGTTTCCAGCATCAT	
AOX3-F1	CGGCACCGAGAAGCATGA	RT-PCR/qRT-PCR analysis of *AOX3* gene
AOX3-R1	CTGGTCCACTTCCACTCCGT	
LBb1.3	ATTTTGCCGATTTCGGAAC	Genotype *rug3* mutants and the complementation lines
Rug3-1-LP	TCCCACCGGTAGACTGTAGTG	
Rug3-1-RP	AACCACCACATGAGACAGAGG	
Rug3-2-LP	ATTGCAAAATCTCTGCCATTG	
Rug3-2-RP	AATCCCATCCTTTTGGTTTTG	
35S-F	GACGCACAATCCCACTATCC	
Dek47-R6	CGAGGAGCGAGGAGTGGAAGAG	
ZmActin-RT-F	TAGTTGAGAATGGCTGACGAGG	RT-PCR, maize RNA normalization
ZmActin-RT-R	ATCTTCAGGCGAAACACGGAGC	
ZmActin-qRT-F	ATGGTCAAGGCCGGTTTCG	qRT-PCR, maize RNA normalization
ZmActin-qRT-R	TCAGGATGCCTCTCTTGGCC	
